# Supplementary material for: Application of Peak Glucose Range and Diabetes Status in Mortality Risk Stratification in Critically Ill Patients with Sepsis
Source: Diagnostics (Basel). 2021 Sep 29;11(10):1798. doi: 10.3390/diagnostics11101798 (PMC8534908; doi:10.3390/diagnostics11101798)
Supplement: Supplementary file 1 [file diagnostics-11-01798-s001.zip › diagnostics-1369235 supplementary.pdf]

Supplementary Material

Evaluation of Peak Glucose Range on Day1 for Predicting Mortality of septic patients in Intensive Care Units

Kai-Yin Hung, Yi-Hsuan Tsai, Chiung-Yu Lin, Ya-Chun Chang, Yi-Hsi Wang, Meng-Chih Lin ,Wen-Feng Fang

Supplementary Materials and Methods

Plasma and purified blood mononuclear cell (PBMC) preparation

Whole blood (20 mL) was collected from the patients in a heparin tube (BD, Franklin Lakes, NJ, USA). The day of ICU admission was defined as day 1. Using a Ficoll-Paque (Amersham Biosciences, Uppsala, Sweden), whole blood was centrifuged at 400 *g* × 30 min to separate the plasma and PBMCs. All PBMC samples were treated immediately, and the plasma samples were stored at -80°C until use. Fresh PBMCs were separated into two aliquots for monocyte human leukocyte antigen-D-related (HLA-DR) expression measurement.

Monocyte HLA-DR expression measurement by flow cytometry

HLA-DR-related monocyte expression was measured by flow cytometry (Cytomics FC500; Beckman Coulter, Inc., Fullerton, CA, USA). Staining and cell acquisition for flow cytometry were performed within 1 h of blood collection. The monoclonal antibodies used were: CD14-PerCP/Cy5.5 (clone: HCD14; Biolegend, San Diego, CA, USA) and HLA-DR-FITC (clone: L243; Biolegend) per 100 µL of PBMCs. The negative controls were mouse monoclonal antibodies IgG1, PerCP/Cy5.5 (clone: MOPC-21), IgG2a, FITC (clone: MOPC-173), and IgG2a, PE (clone: MOPC-173), all of which were isotype-matched, as recommended by the manufacturer. Monocytes were characterised based on the basis of their CD14 expression. At least 30,000 PBMCs were analysed for each sample. The results are expressed as percentages of HLA-DR-positive monocytes per total monocyte population.

Supplemental material for Tables 1

Pairwise comparison:

Significant difference between groups (1=P1 group, 2=P2 group, 3=P3)

7-day mortality: 2 vs. 3

90-day mortality: 2 vs. 3, 1 vs. 3

Age: 1 vs. 3

Body mass index (BMI): 1 vs. 3, 2 vs. 3

Sex: 3 vs. 1, 3 vs. 2

Acute Physiology and Chronic Health Evaluation II (APACHE II): 1 vs. 3, 2 vs. 3

Charlson comorbidity index: nil

Hypertension: 1 vs. 3

Diabetes mellitus: 1 vs. 2, 1 vs. 3, 2 vs. 3

Glycated haemoglobin (HbA1c): 1 vs. 2, 1 vs. 3, 2 vs. 3

Glucose in the emergency room (ER): 1 vs. 2, 1 vs. 3, 2 vs. 3

Table S1 Baseline characteristics and outcomes of septic patients in the validation cohort

by DM status or peak glucose level on day 1	DM (n=199)	Non-DM (n=293)	P†	P1 group (n=153)	P2 group (n=149)	P3 group (n=137)	P*
**Demographic characteristics, median (25th and 75th percentile)**							
Age (years)	70(62.5,79.5)	68(57.5,80.3)	0.104	68(58,79)	68(61,80)	75(64,82)	0.037
BMI, kg/m^2	23.8(20.8,28.2)	22.2(19.5,26.0)	0.001	22.8(19.6,26.6)	23.1(20.1,26.3)	23.7(20.1,27.6)	0.267
Sex (male), n (%)	114 (57.3)	185 (63.1)	0.192	102(38.8)	89(33.8)	72(27.4)	0.050
APACHE II	23(19,28)	21(17,27)	0.001	21(17,26.5)	22(17,27)	23.5(19,28)	0.028
Charlson comorbidity index	5(4,7)	4(2,6)	<0.001	4(2,7)	5(3,7)	6(4,7)	0.005
**Comorbidities, n (%)**							
Coronary artery disease	38 (19.1)	25 (8.5)	0.001	16(27.1)	23(39.0)	20(33.9)	0.399
History of stroke	28 (14.1)	24 (8.2)	0.037	16(37.2)	9(20.9)	18(41.9)	0.124
Hypertension	144 (72.4)	125 (42.7)	<0.001	76(31.0)	78(31.8)	91(37.1)	0.009
COPD	32 (16.1)	44 (15.0)	0.749	23(34.8)	22(33.3)	21(31.8)	0.991
Cancer	42 (21.1)	99 (33.8)	0.002	51(40.2)	41(32.3)	35(27.6)	0.310
CKD	86 (43.4)	55(18.8)	<0.001	38(27.9)	47(34.6)	51(37.5)	0.073
Liver cirrhosis	11 (5.5)	28 (9.6)	0.105	11(29.7)	10(27.0)	16(43.2)	0.254
**Site of suspected infection, n (%)**							
Lung	153 (76.9)	230 (78.5)	0.672	121(35.4)	111(32.5)	110(32.2)	0.454
UTI	131 (65.8)	200 (68.3)	0.573	102(34.9)	95(32.5)	95(32.5)	0.607
Bacteremia	56 (28.1)	90 (30.7)	0.539	54(40.9)	42(31.8)	36(27.3)	0.205

Others	17 (8.5)	21 (7.2)	0.575	14(38.9)	11(30.6)	11(30.6)	0.852
**Mortality, n (%)**							
7-day mortality	9 (4.5)	16 (5.5)	0.642	8(33.3)	7(29.2)	9(37.5)	0.776
28-day mortality	41 (20.6)	60 (20.5)	0.973	33(35.9)	24(26.1)	35(38)	0.143
90-day mortality	74 (37.2)	100 (34.1)	0.486	50(31.4)	48(30.2)	61(38.4)	0.051

† Comparison analyses between two groups by Mann–Whitney U tests or chi-square tests for categorical variables

P*: Comparison analyses among three groups using one-way analysis of variance (ANOVA), with Kruskal–Wallis test as a non-parametric alternative to ANOVA for non-normally distributed continuous variables or chi-square tests for categorical variables

Abbreviations: COPD, chronic obstructive pulmonary disease; CKD, chronic kidney disease; UTI, urinary tract infection; HLA-DR, human leukocyte antigen-D-related

Table S2. Mortality comparisons between DM and non-DM groups by Cox proportional-hazards regression in the validation cohort

			**Model 1**		**Model 2**	
**DM compared to non-DM (non -DM as reference)**	**Crude Hazard Ratio (95% CI)**	**p value**	**Adjusted Hazard Ratio (95% CI)**	**p value**	**Adjusted Hazard Ratio (95% CI)**	**p value**
**Cox proportional-hazards regression analysis**						
7-day mortality	1.224(0.541-2.770)	0.627	1.624(0.665-3.970)	0.287	1.087(0.397-2.975)	0.871
28-day mortality	0.981(0.659-1.459)	0.924	1.248(0.823-1.892)	0.297	0.889(0.557-1.418)	0.622
90-day mortality	0.908(0.672-1.226)	0.529	1.220(0.890-1.672)	0.216	0.822(0.575-1.176)	0.284

Model 1: Adjusted for age, sex, BMI, APACHE II, Charlson comorbidity index

Model 2: Adjusted for age, sex, BMI, APACHE II, Charlson comorbidity index, coronary artery disease, history of stroke, hypertension, COPD, cancer, CKD, liver cirrhosis

Table S3. Cox proportional-hazards regression analysis for 90-day mortality (Crude HR) in the construction cohort

**variable**	**Crude Hazard Ratio (95% CI)**	**p value**
Peak glucose on day 1	1.001 (1.000-1.002)	0.032
DM status (with compared to without)	0.775 (0.621-0 .969)	0.025
3-group tool (P2 compared to P3)<br>(peak glucose level on day 1)	0.680 (0.522-0.884)	0.004
Age	0.998 (0.991-1.005)	0.581
Gender (male)	1.150 (0.924-1.432)	0.212
BMI	0.993 (0.971-1.014)	0.500
APACHE II	1.026 (1.013-1.039)	<0.001
Charlson comorbidity index	1.176 (1.119-1.236)	<0.001
Day 1 SIRS	1.156 (1.041-1.283)	0.006
Day 1 qSOFA	1.275 (1.094-1.485)	0.002
Day 1 SOFA	1.105 (1.075-1.137)	<0.001
Day 3 SIRS	1.317 (1.183-1.466)	<0.001
Day 3 qSOFA	1.568 (1.305-1.884)	<0.001
Day 3 SOFA	1.169 (1.132-1.206)	<0.001
Day 1 HLA-DR expression%	0.983 (0.969-0.997)	0.015
Day 3 HLA-DR expression %	0.979 (0.963-0.995)	0.010
Coronary artery disease	0.854 (0.664-1.097)	0.216
History of stroke	0.822 (0.621-1.089)	0.172

Hypertension	0.853 (0.689-1.056)	0.144
COPD	0.969 (0.720-1.306)	0.838
Cancer	2.105 (1.682-2.635)	<0.001
CKD	1.090 (0.834-1.424)	0.530
Liver cirrhosis	1.824 (1.325-2.513)	<0.001

Table S4. Cox proportional-hazards regression analysis for 90-day mortality (Crude HR) in the validation cohort

variable	Crude Hazard Ratio (95% CI)	p value
Peak glucose on day 1	1.002 (1.000-1.003)	0.015
DM status (with compared to without)	0.908(0.672-1.226)	0.529
3-group tool (P2 compared to P3)<br>(peak glucose level on day 1)	0.648(0.444-0.947)	0.025
Age	1.002(0.992-1.012)	0.731
Gender (male)	0.971(0.715-1.320)	0.853
BMI	0.999(0.992-1.005)	0.633
APACHE II	1.067(1.047-1.088)	<0.001
Charlson comorbidity index	1.141(1.084-1.201)	<0.001
Day 1 SIRS	1.494(1.280-1.744)	<0.001
Day 1 qSOFA	1.587(1.300-1.938)	<0.001
Day 1 SOFA	1.133(1.094-1.174)	<0.001
Day 3 SIRS	1.611(1.394-1.862)	<0.001
Day 3 qSOFA	1.868(1.514-2.304)	<0.001
Day 3 SOFA	1.169(1.128-1.210)	<0.001
Coronary artery disease	1.125(0.706-1.792)	0.621
History of stroke	0.807(0.482-1.350)	0.414
Hypertension	0.850(0.631-1.144)	0.284
COPD	0.889(0.582-1.358)	0.587

Cancer	2.598(1.927-3.503)	<0.001
CKD	0.992(0.714-1.378)	0.961
Liver cirrhosis	2.334(1.505-3.621)	<0.001

Table S5. Cox proportional-hazards regression analysis for 90-day mortality (Adjusted HR) in the validation cohort

	**Model 1**		**Model 2**	
**variable**	**Adjusted Hazard Ratio (95% CI)**	**p value**	**Adjusted Hazard Ratio (95% CI)**	**p value**
**Total**				
Peak glucose on day 1	1.001(0.999-1.003)	0.202	1.002(1.000-1.004)	0.026
DM status (with compared to without)	0.820(0.598-1.123)	0.216	1.216(0.851-1.738)	0.284
3-group tool (P2 compared to P3) (peak glucose level on day 1)	0.758(0.514-1.117)	0.161	0.649(0.429-0.981)	0.040
**With DM**				
Peak glucose on day 1	1.003(1.001-1.005)	*0.005*	1.003(1.001-1.005)	0.005
3-group tool (P2 compared to P3) (peak glucose level on day 1)	0.562(0.325-0.972)	*0.039*	0.511(0.291-0.896)	0.019
**Without DM**				
Peak glucose on day 1	0.999(0.996-1.003)	0.681	0.998(0.995-1.002)	0.347
3-group tool (P2 compared to P3) (peak glucose level on day 1)	1.004(0.553-1.972)	0.894	1.552(0.723-3.330)	0.259

Model 1: Adjusted for age, sex, BMI, APACHE II, Charlson comorbidity index

Model 2: Adjusted for age, sex, BMI, APACHE II, Charlson comorbidity index, coronary artery disease, history of stroke, hypertension, COPD, cancer, CKD, liver cirrhosis

Table S6. Serial clinical sepsis scores and immune status of septic patients (median [IQR]) in the construction cohort

	DM	Non-DM	p†	P1 group	P2 group	P3 group	P*
**Day 1**							
	(n=386)	(n=336)		(n=213)	(n=274)	(n=235)	
SIRS	2 (1)	2 (1)	0.590	2 (1)	2 (1)	3 (1)	0.019
qSOFA	2 (1)	2 (1)	0.014	2 (1)	2 (1)	2 (1)	0.426
SOFA	9 (5)	9 (6)	0.396	9 (5)	8 (5)	10 (6)	0.008
SOFA sub-scores							
Respiratory score	3 (1)	2 (2)	0.933	2 (2)	2 (2)	3 (1)	0.067
CV score	0 (3)	0 (4)	0.253	1 (4)	0 (1)	0 (4)	0.006
CNS score	3 (2)	3 (2)	0.009	3 (2)	3 (2)	3 (2)	0.005
Renal score	1 (3)	1 (2)	0.001	1 (2)	1 (2)	2 (3)	0.084
Coagulation score	0 (2)	0 (2)	0.258	0 (2)	0 (1.5)	0 (2)	0.938
Hepatic score	0 (0)	0 (1)	0.059	0 (1)	0 (0)	0 (1)	0.313
HLA-DR expression (%)#	92.4 (21.1)	91.9 (19.8)	0.699	90.4 (24)	96.6 (13.7)	88.8 (21.7)	0.013
**Day 3**							
	(n=368)	(n=306)		(n=196)	(n=262)	(n=216)	
SIRS	2 (2)	2 (2)	0.825	2 (2)	2 (2)	2 (2)	0.710
qSOFA	1 (1)	1 (1)	0.143	1 (1)	1 (1)	1 (1)	0.774
SOFA	7 (5)	7 (5)	0.785	8 (5)	7 (5)	7 (5)	0.330
SOFA sub-scores							
Respiratory score	2 (1)	2 (2)	0.090	2 (2)	2 (2)	2 (1)	0.109

CV score	0 (1)	0 (1)	0.159	0 (1)	0 (1)	0 (1)	0.359
CNS score	3 (1)	3 (1)	0.233	3 (1)	3 (1)	3 (2)	0.013
Renal score	1 (3)	0 (2)	0.004	1 (2)	1 (3)	1 (3)	0.110
Coagulation score	1 (2)	1 (2)	0.382	1 (2)	0 (2)	1 (2)	0.275
Hepatic score	0 (1)	0 (1)	0.043	0 (1)	0 (1)	0 (1)	0.719
HLA-DR expression (%)	93.3 (14.3)	94.9 (14.0)	0.242	92.9 (13.6)	97.6 (9.1)	91.1 (16.7)	0.001

Expressed as median (interquartile range)

Patients with HLA-DR data on day 1: DM=89, non-DM =60, P1=38, P2=52, P3=59

Patients with HLA-DR data on day 3: DM=82, Non-DM=53, P1=37, P2=48, P3=50

† Comparison analyses between two groups by Mann–Whitney U tests or chi-square tests for categorical variables

P*: Comparison analyses among three groups using one-way analysis of variance (ANOVA), with Kruskal–Wallis as a non-parametric alternative to ANOVA for non-normally distributed continuous variables or chi-square tests for categorical variables

Abbreviations: HLA-DR, human leukocyte antigen-D-related

Table S7. Serial clinical sepsis scores and immune status of septic patients (median [IQR]) in the validation cohort

	DM	Non-DM	p[†]	P1 group	P2 group	P3 group	P*
**Day 1**							
	(n=199)	(n=293)		(n=153)	(n=149)	(n=137)	
SIRS	2(2)	2(1)	0.190	2(1)	2(2)	2(2)	0.427
qSOFA	1(1)	1(1)	0.117	1(1)	1(1)	1.5(1)	0.915
SOFA	10(6)	9(7)	0.861	8(6)	9(6)	10.5(7)	0.226
Respiratory score	3(3)	3(2)	0.566	2(2)	3(2)	3(3)	0.465
CV score	0(0)	0(4)	0.873	0(4)	0(0)	0(4)	0.308
CNS score	2(2)	2(1)	0.010	2(1)	2(1)	3(2)	0.509
Renal score	2(2)	1(2)	<0.001	1(2)	1(3)	2(3)	<0.001
Coagulation score	0(2)	0(1)	0.631	0(2)	0(1)	0(2)	0.245
Hepatic score	0.5(2)	0(2)	0.712	0(1)	0(2)	1(2)	0.366
**Day 3**							
	(n=199)	(n=293)		(n=153)	(n=149)	(n=137)	
SIRS	2(2)	2(1)	0.228	1.5(1)	2(2)	2(2)	0.141
qSOFA	1(1)	1(1)	0.832	1(1)	2(1)	1(1)	0.910
SOFA	7(6)	7(5)	0.300	6(5)	7(6)	7.5(7)	0.510
Respiratory score	2(2)	2(2)	0.199	1.5(2)	2(1)	2(2)	0.582
CV score	0(0)	0(3)	0.696	0(3)	0(0)	0(1)	0.084

CNS score	2(2)	2(1)	0.835	2(1)	2(1)	2(1)	0.840
Renal score	2(3)	0(2)	<0.001	1(2)	1(3)	1(2)	0.312
Coagulation score	1(2)	0(2)	0.024	0(2)	1(2)	1(2)	0.137
Hepatic score	0(1)	0(1)	0.061	0(1)	0(1)	1(2)	0.796

Expressed as median (interquartile range)

† Comparison analyses between two groups by Mann–Whitney U tests or chi-square tests for categorical variables

P*: Comparison analyses among three groups using one-way analysis of variance (ANOVA), with Kruskal–Wallis as a non-parametric alternative to ANOVA for non-normally distributed continuous variables or chi-square tests for categorical variables

Table S8. Baseline characteristics and outcomes of septic patients between cohorts

	Construction cohort (n=722)	Validation cohort (n=492)	P†
**Demographic characteristics, median (25th and 75th percentile)**			
Age (years)	68 (59.0,79.0)	69.2 (59.5,80.1)	0.053
BMI, kg/m^2	22.5 (19.4,25.9)	23.0 (19.8,26.6)	0.068
Sex (male), n (%)	431 (59.7)	299 (60.8)	0.707
APACHE II	25 (20,31)	22 (17,27)	<0.001
Charlson comorbidity index	2 (1,3)	5 (3,7)	<0.001
**Comorbidities, n (%)**			
Coronary artery disease	187 (25.9)	63 (12.8)	<0.001
History of stroke	133 (18.4)	52 (10.6)	<0.001
Hypertension	409 (56.7)	269 (54.7)	0.480
COPD	101 (14.0)	76 (15.4)	0.480
Cancer	166 (23.2)	141 (28.7)	0.033
CKD	229 (31.7)	141 (28.7)	0.265
Liver cirrhosis	68 (9.4)	39 (7.9)	0.368
**Site of suspected infection, n (%)**			
Lung	458 (63.4)	383 (77.8)	<0.001
UTI	157 (21.7)	331 (67.3)	<0.001
Bacteremia	57 (7.9)	146 (29.7)	<0.001

Others	146 (20.2)	38 (7.7)	<0.001
**Mortality, n (%)**			
7-day mortality	98 (13.6)	25 (5.1)	<0.001
28-day mortality	212 (29.4)	101 (20.5)	0.001
90-day mortality	311 (43.1)	174 (35.4)	0.007

Table S9. Baseline characteristics and outcomes of septic patients between study and exclusion group in construction cohort

by DM status or peak glucose level on day 1	Study group (n=722)	Exclusion group (n=77)	P[†]
**Demographic characteristics, median (25th and 75th percentile)**			
Age (years)	68.0 (59.0-79.0)	65.0 (54.0-76.0)	0.234
BMI, kg/m^2	22.5 (19.4-25.9)	21.9 (18.8-24.6)	0.054
Sex (male), n (%)	431 (58.9)	45 (584)	0.831
APACHE II	25.0 (20.0-31.0)	20.0 (15.0-25.0)	<0.001
Charlson comorbidity index	2.0 (1.0-3.0)	2.0 (1.0-6.0)	0.356
**Comorbidities, n (%)**			
Coronary artery disease	187 (25.9)	17 (22.1)	0.465
History of stroke	133 (18.4)	14 (18.2)	0.959
Hypertension	409 (56.7)	35 (45.5)	0.058
COPD	101 (14.0)	16 (20.8)	0.109
Cancer	166 (23.2)	24 (31.3)	0.121

CKD	160 (31.6)	6 (11.3)	0.002
Liver cirrhosis	68 (9.4)	1 (1.3)	0.016
Diabetes mellitus	349 (48.3)	7 (9.1)	<0.001
**Site of suspected infection, n (%)**			
Lung	458 (63.1)	57 (74.0)	0.065
UTI	157 (21.7)	12 (15.6)	0.208
Bacteremia	57 (7.9)	4 (5.2)	0.396
Others	146 (20.2)	13 (16.9)	0.485
**Baseline glucose and HbA1c, median (25th and 75th percentile)**			
HbA1c (%)	6.7(5.8-8.0)	6.0(5.6-6.7)	0.017
Glucose (mg/dL)	159.0(125.0-243.0)	143.5(118.8-192.8)	0.026
**Mortality, n (%)**			
7-day mortality	98 (13.6)	4 (5.2)	0.036
28-day mortality	212 (29.4)	15 (19.5)	0.068
90-day mortality	311 (43.1)	28 (36.4)	0.257

stratified by DM status or peak glucose level on day 1

† Comparison analyses between two groups by Mann–Whitney U tests or chi-square tests for categorical variables
